# A Follow-Up Study on Testicular Cellular and Transcriptomic Responses to Mild Scrotal Heat Stress in Wugu-Hu and Hu Rams

**DOI:** 10.3390/ani16091317

**Published:** 2026-04-25

**Authors:** Shikun Chen, Qingjie Pan, Henry Annandale, Peter Charles Irons, Huansheng Dong

**Affiliations:** 1 College of Animal Science and Technology, Qingdao Agricultural University, Qingdao 266109, China; 34095258@student.murdoch.edu.au (S.C.);; 2 College of Environmental and Life Sciences, Murdoch University, Murdoch, Perth, WA 6150, Australia

**Keywords:** scrotal heat stress, transcriptomic analysis, ram, testicular cells

## Abstract

This study aimed to describe the endocrine, cellular, and transcriptomic changes associated with mild heat stress responses in Wugu-Hu and Hu rams. Testicular samples from rams exposed to 3 days of scrotal insulation, resulting in an approximate 3 °C increase in scrotal temperature, and corresponding controls were analyzed for endocrine activity, seminiferous tubule morphology, germ cell composition, and transcriptomic profiles. There were no significant changes in testosterone, follicle-stimulating hormone, or luteinizing hormone after 3 days of mild heat exposure in either breed. Wugu-Hu rams showed greater disorganization of seminiferous tubules. Apoptotic events occurred mainly from spermatocytes to spermatids and were accompanied by a greater decline in spermatids in Wugu-Hu rams. Comparative transcriptomic analysis between Wugu-Hu and Hu rams identified 854 differentially expressed genes, mainly enriched in immune response function. We conclude that scrotal heat stress does not disrupt endocrine balance at the level applied in this study, but it induces breed-dependent morphological and testicular cellular responses. The differences in the immune response of Hu and Wugu-Hu rams may contribute to their distinct levels of spermatocytes and spermatids’ response to elevated temperatures.

## Simple Summary

This study investigated the impact of a brief period of mild heat exposure on fertility-related cells in two types of rams. We aimed to understand which testicular cells are most vulnerable, whether the Wugu-Hu rams and Hu rams respond differently, and whether their hormone levels or gene activity help explain these differences. We found that heat did not disturb their overall reproductive hormone balance. However, one breed showed more obvious structural damage in the testicular tissue and a greater loss of developing sperm cells. When we compared gene activity between the two breeds, we found clear differences in genes linked to the body’s defence and stress responses, which may help explain why they react differently to heat.

## 1. Introduction

Mammalian testis function is highly temperature-dependent [1,2,3,4]. Increased temperature of the scrotum and testes can harm the testicular cells [5,6]. Even an increase of approximately 1–2 °C in scrotal temperature can disrupt spermatogenesis, impair sperm quality, and reduce fertility in mammals [7,8]. In rams, reproductive performance is susceptible to heat stress, as elevated environmental temperatures can challenge thermoregulatory capacity and adversely affect testicular function and spermatogenesis [9].

Studies have shown that heat stress can cause oxidative damage, DNA damage, and cell death in male germ cells [7,10,11,12]. Evidence suggests that spermatogonia have a relatively high capacity for self-repair; germ cells at late developmental stages often fail to recover from injury, leading to germ cell loss and reduced sperm quality [5,13,14,15]. Recent studies have further demonstrated that the severity of these effects depends not only on the intensity and duration of heat exposure but also on breed-specific physiological and cellular characteristics [16]. A previous study demonstrated that Wugu-Hu rams exhibited stronger responses to mild scrotal heat stress than Hu rams, including more extensive transcriptomic changes in testicular cells and potential impairment of meiotic processes, while maintaining semen quality through enhanced apoptotic clearance of damaged cells [16]. While many mechanistic insights into germ cell damage are derived from rodent models, species-specific differences in testicular physiology should be considered, and these findings may not fully translate to ruminants such as sheep. It remains unclear which specific germ cell populations are most affected and how breed-dependent gene expression contributes to cellular heat tolerance.

Therefore, the present study builds on a previously published study using the same scrotal insulation model, in which continuous monitoring of scrotal temperature and the temperature humidity index (THI) confirmed that the model induced a stable mild heat stress condition [16]. Based on previous evidence regarding breed-dependent testicular cell apoptosis following mild scrotal heat stress, we hypothesized that mild heat stress induces cell-type-specific alterations within the seminiferous epithelium and that these alterations are more pronounced in Wugu-Hu rams than in Hu rams, particularly in germ cells at meiotic and post-meiotic stages. To test this hypothesis, we combined quantitative analyses of seminiferous tubule morphology, Sertoli cells, spermatogonia, spermatocytes, and spermatids with comparative transcriptomic profiling between the two breeds. This integrated approach provides new insight into how breed-dependent cellular and molecular characteristics influence testicular cell response to mild heat stress.

## 2. Materials and Methods

### 2.1. Testicular Samples

This study was conducted using the same testicular tissue samples as described in Chen, Jiang, Wang, Pan, Annandale, Irons, and Dong’s [16] study. In short, in this experiment, three Wugu-Hu rams and three Hu rams in the treatment group were subjected to scrotal insulation for 3 consecutive days to simulate mild heat stress conditions. Scrotal insulation was applied using a validated experimental model as described previously [16], which has been shown to increase testicular temperature by approximately 3 °C without inducing systemic hyperthermia. The insulation protocol was applied consistently across all animals under identical environmental conditions. Testicular samples were collected from three Hu rams following scrotal insulation and from three Hu rams from a control group, as well as from scrotal insulation and control groups of Wugu-Hu sheep (*n* = 3 per group), totaling 12 rams. The groups were named Hu sheep scrotal insulation (post-insulation), Hu sheep control (pre-insulation), Wugu-Hu sheep scrotal insulation (post-insulation) and Wugu-Hu control (pre-insulation), respectively. Adult rams 1.5 to 2 years of age managed in purpose-built housing on a commercial farm in Rizhao, China, were used. All experimental animals were provided with identical nutrition and unrestricted access to clean drinking water, and they were housed under uniform environmental conditions throughout the study [16]. The rams were then slaughtered, and testicular tissue was harvested.

Testicular samples were collected immediately after the 3-day scrotal insulation, without a recovery interval, to capture the direct effects of mild heat stress on testicular cellular composition and morphology. The testicular samples included Hu sheep and Wugu-Hu sheep from the scrotal insulation treatment groups (post-insulation) and non-insulated control groups (pre-insulation). Tissue blocks were cut to approximately 10 mm × 10 mm × 5 mm.

All animal procedures were conducted according to the ethical guidelines for the care and use of animals for scientific purposes. The Animal Ethics Committee at Murdoch University reviewed and approved the study (Approval Code: OS3447/23, Approval Date: 12 September 2023).

### 2.2. Hormone Assays

Testosterone, LH, and FSH concentrations were assayed in testicular tissue from Hu and Wugu-Hu sheep using ELISA kits purchased from Jiancheng (Nanjing, China), including an FSH kit (Cat. H101-1-2), an LH kit (Cat. H206-1-2), and a testosterone kit (Cat. H090-1-2). Briefly, 20 mg of testicular tissue was homogenized in 1 mL of physiological saline and centrifuged at 3000 rpm for 10 min, and the supernatant was collected for the analysis. For each assay, 50 μL of standard solution was added to the standard wells, while 10 μL of sample and 40 μL of sample diluent were added to the sample wells. Blank wells contained no sample or standard. Then, 100 μL of HRP conjugate reagent was added to each well, followed by incubation for 60 min at 37 °C. Wells were washed five times, after which 50 μL of chromogen solution A and 50 μL of chromogen solution B were added and incubated for 15 min at 37 °C in the dark. The reaction was terminated by adding 50 μL of stop solution, and optical density was measured at 450 nm within 15 min using a Tecan microplate reader (SN2008010265, Tecan Group Ltd., Männedorf, Switzerland).

### 2.3. Hematoxylin and Eosin Staining

Testicular tissue blocks were fixed in 4% paraformaldehyde solution (Fuyu, Tianjin, China). After 12 h, the paraformaldehyde-fixed tissues were sequentially dehydrated in 30%, 50%, 75%, 90%, 95%, 100% (I), and 100% (II) alcohol (Fuyu, Tianjin, China), with each gradient lasting 30 min. The tissue was then placed in xylene (Fuyu, Tianjin, China) for 20 min before being immersed in paraffin (Fuyu, Tianjin, China) with a melting point of 56 °C for 12 h. Finally, the tissue was embedded in paraffin for sectioning, and slices were prepared using a paraffin rotary slicer (SN02026, Leica Microsystems, Shanghai, China) with a thickness of 4 μm.

Paraffin sections were baked at 60 °C for 60 min, they were deparaffinised in xylene twice for 10 min each, and then they were rehydrated through a graded ethanol series before rinsing in ddH_2_O. the sections were stained with hematoxylin (Solarbio, Beijing, China) for 2 min, rinsed thoroughly with tap water, differentiated in 1% HCl in 70% ethanol (Fuyu, Tianjin, China) for 3 s, and rinsed again under running tap water for 2 min. Finally, the sections were counterstained with eosin (Solarbio, Beijing, China) for 1 min and rinsed thoroughly with tap water.

### 2.4. Immunofluorescence and Fluorescent Staining

DAPI (Cat.C0065, Solarbio, Beijing, China) and PNA (Solarbio, Beijing, China) fluorescence were used to demonstrate nuclear localization and to identify spermatids within the seminiferous tubules, respectively. For immunofluorescence, the dewaxing and rehydration were consistent with the above methods. For antigen retrieval, the sections were boiled in 0.1 M citrate buffer (Solarbio, Beijing, China) for 20 min, and then they were cooled naturally to room temperature. The sections were washed with phosphate-buffered saline (PBS) three times, 5 min each time. After washing, 10% bovine serum albumin (BSA) (Cat.A8020, Solarbio, Beijing, China) was applied onto the sections and blocked at room temperature for 60 min. The blocking solution was removed without washing. The primary antibodies were added, namely SOX9 (rabbit monoclonal antibody, 1:200; Abclonal, Wuhan, China) for Sertoli cells and UCLH1 for spermatogonia (mouse monoclonal antibody, 1:100; Thermo Fisher Scientific, USA), at the recommended dilution, ensuring coverage over the sections. The sections were placed in a humidified chamber and incubated overnight at 4 °C. After primary antibody incubation, the sections were washed with PBS three times for 5 min each in a dark environment. Finally, DAPI was applied as a nuclear counterstain and incubated in the dark for 1 min at room temperature. The sections were gently washed with PBS twice for 2 min to remove excess nuclear stain. They were then mounted and immediately observed under a fluorescence microscope for imaging.

For PNA fluorescent staining, the same steps as above, up to the antigen retrieval, process were followed. After antigen retrieval, the sections were washed with PBS, then Alexa Fluor 488-PNA (peanut agglutinin) was applied. The sections were incubated overnight at 4 °C to specifically label spermatids. After incubation, the sections were washed with PBS, and nuclear counterstaining was subsequently performed using DAPI for 30 s in the dark at room temperature. Finally, the sections were mounted and observed under a fluorescence microscope for imaging.

### 2.5. Microscopy

Immunohistochemistry (IHC) and immunofluorescence images were collected under a microscope (Carl-Zeiss GmbH, Jena, Germany) at 200× and 400× magnifications. To ensure consistent colour presentation across all IHC-stained tissue sections, we performed colour normalization using Adobe Photoshop 2024 (Adobe Inc., San Jose, CA, USA).

### 2.6. RNA Sequencing and Bioinformatic Analysis

The raw RNA-seq data used in this study were obtained from our previously published dataset [16]. However, in the present work, the dataset was re-analyzed using different comparison groups and analytical objectives to address distinct biological questions. The differential expression analysis in the present study primarily focused on comparisons between breeds under controlled conditions (Hu pre-insulation and Wugu-Hu pre-insulation) to investigate transcriptomic differences. In short, total RNA was extracted from the testicular tissue samples using the TRIzol reagent (Solarbio, Beijing, China) according to the manufacturer’s instructions. RNA-seq libraries were constructed from testicular tissue samples collected from individual animals, with each biological replicate representing one animal (*n* = 3 per group). RNA purity and concentration were assessed using a spectrophotometer, and RNA integrity was evaluated using an Agilent 2100 Bioanalyzer. Only samples with RNA integrity number ≥ 7.0 were used for library construction. RNA-seq libraries were prepared using a standard Illumina-compatible library preparation protocol and sequenced on an Illumina platform to generate 150 bp paired-end reads. Raw sequencing reads were subjected to quality control to remove adapter sequences, low-quality reads, and reads containing excessive ambiguous bases. Clean reads passing quality filtering were retained for downstream analysis. The filtered reads were mapped to the reference genome of sheep (*Ovis aries* Linnaeus, 1758), and gene expression levels were quantified as fragments per kilobase of transcript per million mapped reads.

A differential expression analysis between groups was performed using DESeq2, which applies a negative binomial distribution model. Genes with an adjusted *p* value ≤ 0.05 were considered differentially expressed. Functional enrichment analyses, including Gene Ontology (GO) and KEGG pathway analyses, were conducted using the clusterProfiler R package.

A protein–protein interaction network (PPI) analysis was performed using the STRING website https://cn.string-db.org/ (accessed on 1 December 2025) with default parameters. Cytoscape software (v3.10.3, Cytoscape Consortium, San Diego, CA, USA) was used to construct and visualize the network relationships of differentially expressed genes.

The RNA-seq experimental design and bioinformatic pipeline were identical to those described in our previous study [16].

### 2.7. Statistical Analysis

For each immunofluorescence group, 100 seminiferous tubule cross-sections from randomly selected slices were counted to quantify the number of SOX9, UCLH1, and PNA positive cells. Spermatocyte numbers were not directly measured at the individual cell level. Instead, they were indirectly derived from average cell counts per seminiferous tubule based on total cell counts and identified cell populations (Sertoli cells, spermatogonia, and spermatids).

Mean_spermatocytes_ = Mean_total_ − (Mean_spermatogonia_ + Mean_spermatids_ + Mean_Sertoli cells_) (1)

(2) $${\mathrm{SD}}_{\mathrm{spermatocytes}}=\sqrt{SD_{total}^{2}+SD_{spermatogonia}^{2}+SD_{spermatids}^{2}+SD_{Sertolicells}^{2}}$$

Quantitative data are expressed as mean cell counts per seminiferous tubule ± standard deviation (SD). Quantification was performed using ImageJ2-Fiji software (version 1.54p; National Institutes of Health, USA). Statistical analyses were conducted using GraphPad Prism (version 10; GraphPad Software, San Diego, CA, USA). For experiments involving two breeds (Hu and Wugu-Hu rams) and two treatment conditions (pre-insulation and post-insulation), the data were analyzed using two-way analysis of variance (ANOVA), with breed and treatment as fixed factors. Interaction effects between breed and treatment were assessed. When significant main effects or interactions were detected, post hoc multiple comparisons were performed using Tukey’s honestly significant difference (HSD) test to adjust for multiple comparisons. Adjusted *p* values were reported for all post hoc analyses. The value of *p* < 0.05 was considered statistically significant. Because spermatocyte counts were indirectly estimated, statistical results related to this parameter were interpreted cautiously.

## 3. Results

### 3.1. Hormonal Changes in the Testes

**Figure 1:**
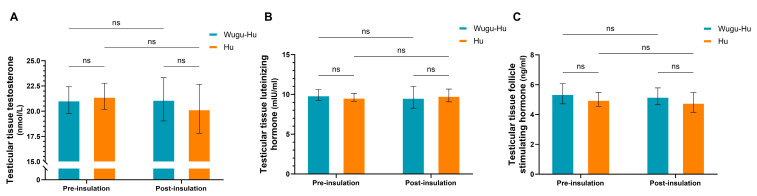
Reproductive hormone concentrations in testicular tissues before and after scrotal insulation in Wugu-Hu crossbred and purebred Hu rams. (**A**) Testosterone, (**B**) LH, (**C**) FSH. Key: not significant (ns) *p* > 0.05.

The results in testicular tissue concentrations of testosterone (Figure 1A), LH (Figure 1B), or FSH (Figure 1C) revealed no significant differences between the two breeds, neither before nor after the insulation treatment (*p* > 0.05). Furthermore, mild scrotal heat stress did not significantly alter the tissue concentrations of these hormones within each breed compared to pre-insulation levels (*p* > 0.05).

### 3.2. Morphology and Quantitative Alterations in Seminiferous Tubules

**Figure 2:**
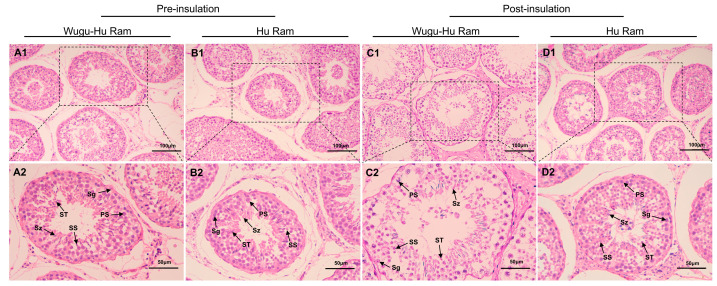
H&E stained sections of testicular tissue showing individual seminiferous tubules in Wugu-Hu and Hu rams pre- and post-scrotal insulation. Legend: Sg: spermatogonium; PS: primary spermatocyte; SS: secondary spermatocyte; ST: spermatid; Sz: sperm. (**A1**–**D1**) Magnification: 400×. (**A2**–**D2**) Scale bars correspond to 25 μm; magnification: 200×.

Figure 2 shows testicular histological sections of Wugu-Hu sheep and Hu sheep pre- and post-insulation. Before insulation, seminiferous tubules in both Wugu-Hu and Hu sheep showed intact and organized structure with spermatogonia, Sertoli cells, spermatocytes, and spermatozoa arranged in a well-organized, stratified manner within the seminiferous tubules (Figure 2(A1,A2,B1,B2)).

After insulation, testicular sections showed varying degrees of structural alterations (Figure 2(C1,C2,D1,D2)). These morphological changes were more marked in Wugu-Hu crossbred sheep, consisting of looser and disorganized seminiferous epithelium and greater luminal enlargement (Figure 2(C2)). Meanwhile, Hu sheep displayed relatively moderate histological alterations, maintaining relatively intact tubular structures despite mild disruptions (Figure 2(D2)).

**Figure 3:**
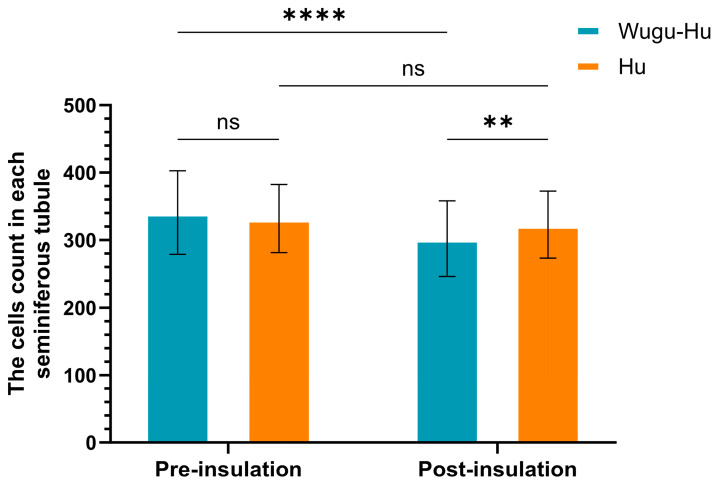
Comparison of total cell counts per seminiferous tubule pre- and post-scrotal insulation in Hu and Wugu-Hu rams. Key: asterisks indicate Tukey-adjusted *p* values. ns *p* > 0.05, ** *p* ≤ 0.01, **** *p* ≤ 0.0001.

The quantitative analysis indicated a significant decrease in the total number of cells per seminiferous tubule in Wugu-Hu sheep post-insulation compared to pre-insulation from 340.67 to 302.12 (*p* < 0.001) (Figure 3). Hu rams did not exhibit a significant change in cell counts following the insulation treatment, from 331.85 to 322.78 (*p* > 0.05).

### 3.3. Quantification of Sertoli Cells

**Figure 4:**
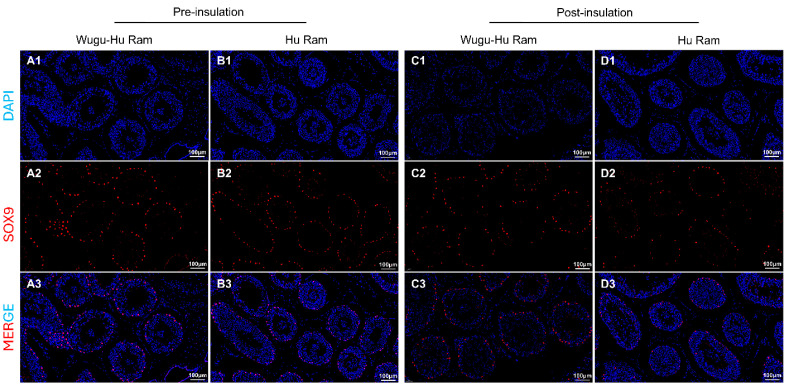

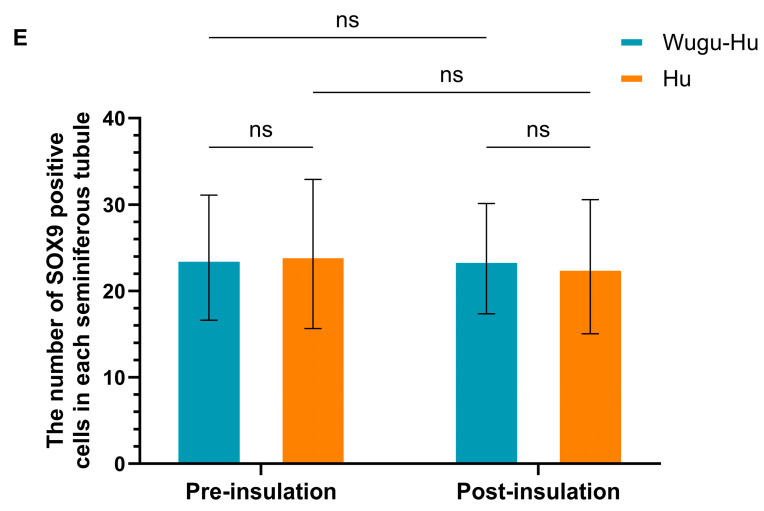
Visualization (**A**–**D**) and quantification (**E**) of SOX9-positive Sertoli cells in seminiferous tubules of Hu and Wugu-Hu rams pre- and post-scrotal insulation. Legend: (**A1**–**D1**) DAPI staining. DAPI (blue) stain cell nuclei. (**A2**–**D2**) SOX9 staining. SOX9 (red) marks Sertoli cells. (**A3**–**D3**) Merged DAPI and SOX9 images. Magnification: 200×. (**E**) Key: ns *p* > 0.05.

Immunofluorescence of Sertoli cells using the antibody UCLH1 is shown in Figure 4(A1–D1,A2–D2,A3–D3). SOX9-positive cells displayed distinct nuclear localization, clearly identifiable within the seminiferous tubules of both sheep breeds, pre- and post-insulation.

The quantitative analysis of SOX9-positive Sertoli cells revealed no significant differences between the two breeds or within each breed when comparing pre- and post-insulation (*p* > 0.05) (Figure 4E). The numbers of Sertoli cells per seminiferous tubule remained consistent and stable across all experimental groups. There was no detectable impact on the abundance of Sertoli cells within the testicular tissue of either sheep breed.

### 3.4. Quantification of Spermatogonia

**Figure 5:**
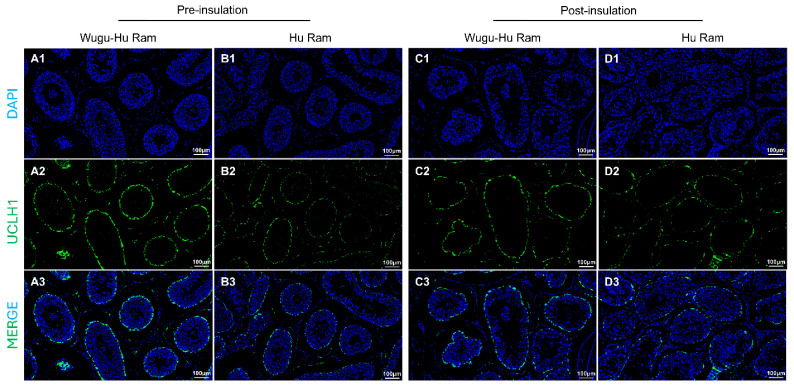

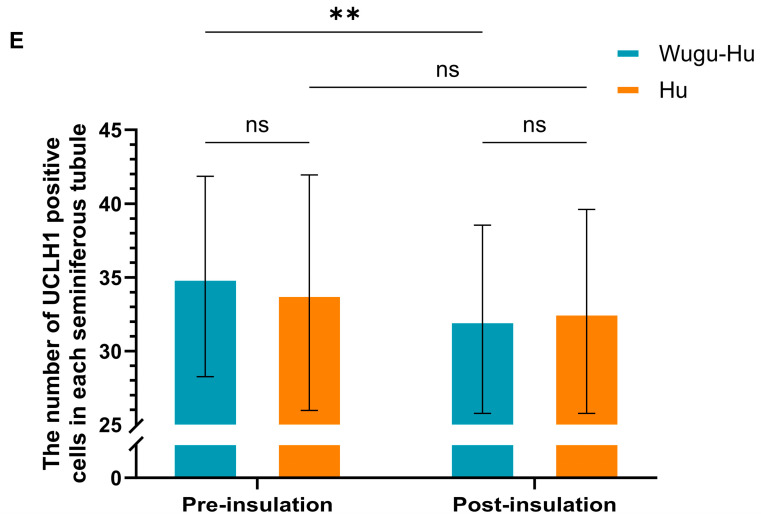
Visualization (**A**–**D**) and quantification (**E**) of UCLH1-positive cells in seminiferous tubules of Hu and Wugu-Hu rams pre- and post-scrotal insulation. Legend: (**A1**–**D1**) DAPI staining. DAPI (blue) stain cell nuclei. (**A2**–**D2**) UCLH1 staining. UCLH1 (green) marks spermatogonia. (**A3**–**D3**) Merged DAPI and UCLH1 images. Magnification: 200×. (**E**) Key: ns *p* > 0.05, ** *p* ≤ 0.01.

Immunofluorescence staining of spermatogonia using the antibody UCLH1 is shown in Figure 5(A1–D1,A2–D2,A3–D3). UCLH1-positive cells showed observed localisation within the seminiferous tubules across both sheep breeds, clearly distinguishing spermatogonia populations.

The quantitative analysis indicated a significant decrease in the number of UCLH1-positive spermatogonia per seminiferous tubule in Wugu-Hu rams post-insulation compared to pre-insulation (*p* < 0.01), but not in Hu rams (*p* > 0.05) (Figure 5E).

### 3.5. Quantification of Spermatids

**Figure 6:**
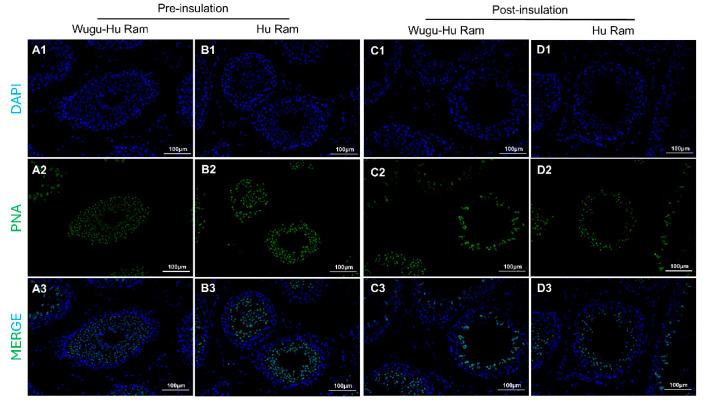

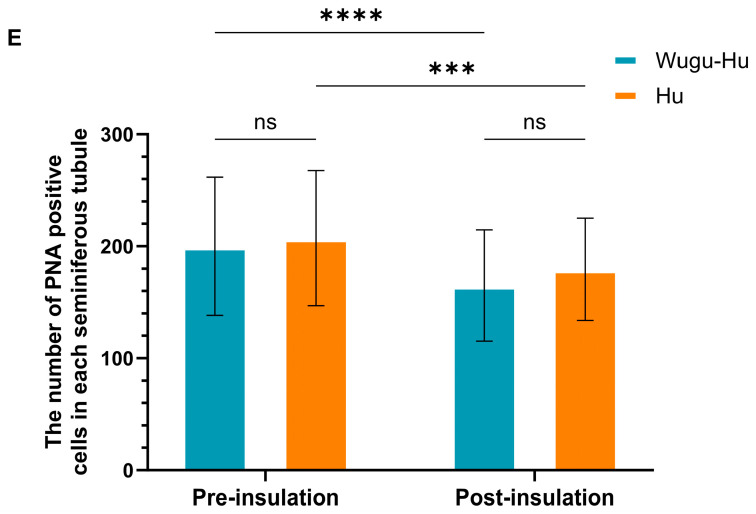
Visualization (**A**–**D**) and quantification (**E**) of PNA positive cells in seminiferous tubules of Hu and Wugu-Hu rams pre- and post-scrotal insulation. Legend: (**A1**–**D1**) DAPI staining. DAPI (blue) stain cell nuclei. (**A2**–**D2**) PNA staining. PNA (green) marks spermatids. (**A3**–**D3**) Merged DAPI and PNA images. Magnification: 200×. (**E**) Key: ns *p* > 0.05; *** *p* ≤ 0.001, **** *p* ≤ 0.0001.

The images of sections of testicular tissues are shown in Figure 6(A1–D1,A2–D2,A3–D3). Spermatids were identified clearly by bright green fluorescence.

The results of spermatid counts are shown in Figure 6E. No significant difference in the number of spermatids per seminiferous tubule was observed between the pre-insulation rams of different breeds (*p* > 0.05). Both Wugu-Hu rams and Hu rams showed a significantly decreased number of spermatids post-insulation compared with pre-insulation (*p* < 0.0001).

### 3.6. Descriptive Estimation of Spermatocyte Abundances

Descriptive estimates of spermatocyte abundance are presented in Table 1. These values were derived indirectly from averaged cell counts per seminiferous tubule rather than being measured directly in individual samples and should therefore be interpreted with caution.

### 3.7. Differentially Expressed Genes of Wugu-Hu Rams vs. Hu Rams

**Figure 7:**
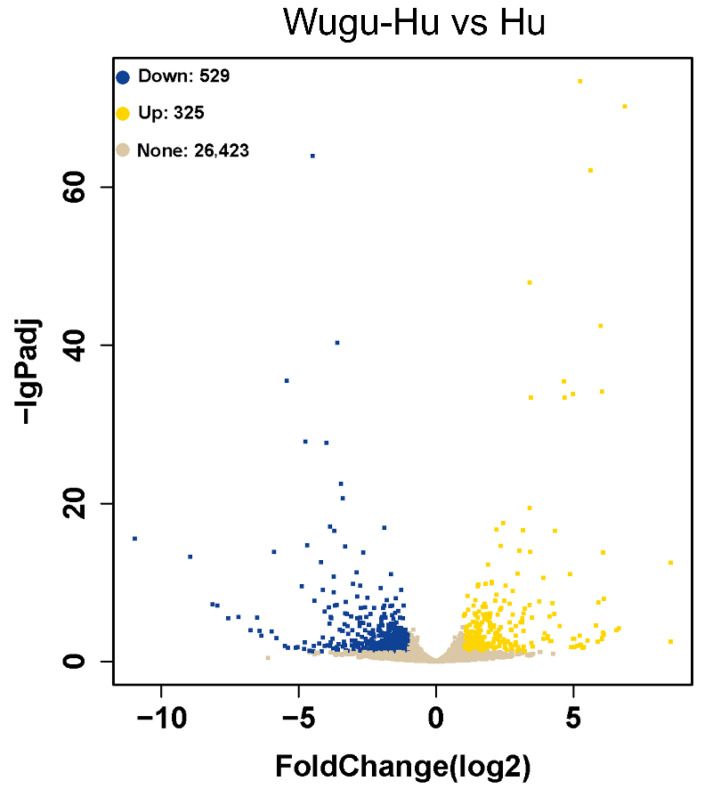
Volcano plot of DEGs between pre-insulation Wugu-Hu and Hu rams. Note: yellow points represent upregulated genes, and blue points represent downregulated genes, *p* ≤ 0.05.

In the comparison between the Wugu-Hu ram and Hu ram groups, 854 DEGs were identified, 325 being upregulated and 529 being downregulated (*p* ≤ 0.05) (Figure 7).

### 3.8. Gene Ontology and KEGG Enrichment

**Figure 8:**
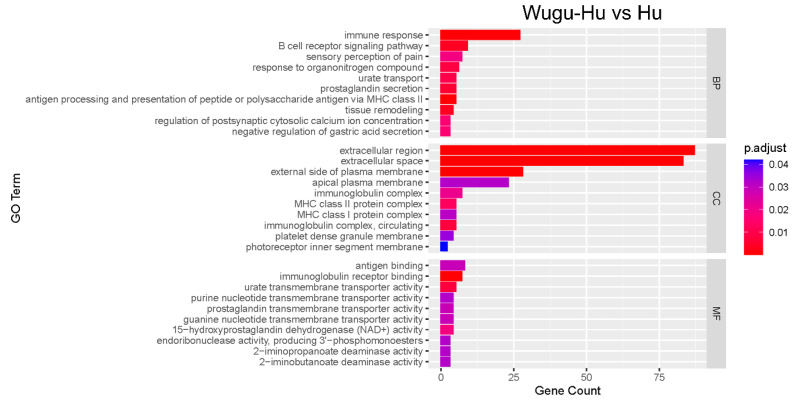
GO enrichment analysis of DEGs between Wugu-Hu and Hu rams. Note: the vertical axis represents GO terms, while the horizontal axis indicates the count of genes enriched in each term relative to the total number of genes. The colour denotes the adjusted *p* value, with deeper red colours signifying higher significance.

The GO analysis was performed on 854 DEGs in the Wugu-Hu vs. Hu group. A total of 697 DEGs were enriched into 56 GO terms (Figure 8). The top three significant enrichments in the biological processes (BP) category are the immune response (27 DEGs), B-cell receptor signalling pathway (nine DEGs), and sensory perception of pain (seven DEGs). The top three significant enrichments in the cellular component (CC) category are the extracellular region (87 DEGs), extracellular space (83 DEGs), and external side of the plasma membrane (28 DEGs). The top three significant enrichments in the molecular function (MF) category are antigen binding (eight DEGs), immunoglobulin receptor binding (seven DEGs), and urate transmembrane transporter activity (five DEGs).

No significantly enriched KEGG pathways were identified based on the current dataset and selection criteria.

### 3.9. Interaction Network Analysis of Proteins

**Figure 9:**
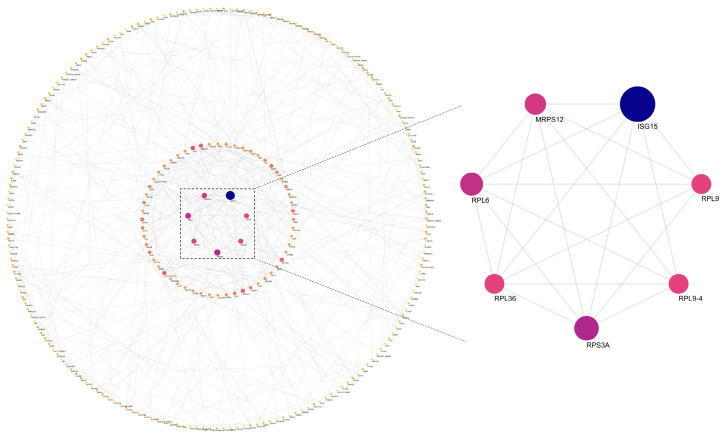
PPI analysis of key proteins in DEGs. Note: nodes with higher degrees of relatedness are larger; darker blue nodes have higher degrees of relatedness, whereas redder nodes have lower degrees of relatedness.

Figure 9 shows the protein–protein interaction (PPI) analysis of DEGs between the Wugu-Hu and Hu rams. This subnetwork included proteins such as RPL6, RPL9, RPL36, RPL9-4, RPS3A, and MRPS12. ISG15 exhibited the highest degree of the module.

## 4. Discussion

This study explored the cellular and transcriptomic mechanisms that regulate testicular responses to mild scrotal heat stress in Hu and Wugu-Hu rams. The findings showed that short-term mild scrotal insulation (~3 °C increase for 3 days) did not affect testicular hormone levels but caused breed-specific morphological and cellular changes within the seminiferous epithelium. Wugu-Hu rams showed greater structural disruption and loss of germ cells, especially at the spermatid stage, whereas Hu rams displayed stronger stability of their germ cells. Transcriptomic analyses suggest that breed-specific differences in immune regulation and translational activity are primarily associated with baseline molecular characteristics of the two breeds, which may in turn influence their adaptive responses to mild heat stress.

### 4.1. Breed-Specific Differences in Spermatogenesis

Breed-dependent variation in heat stress tolerance has been widely reported in sheep, which helps interpret the present findings. Comparative studies have demonstrated that Dorper sheep exhibit relatively higher tolerance to thermal challenges than several temperate or crossbred sheep, as reflected by smaller increases in body temperature and metabolic disruption under heat stress [17]. Additionally, tropical and indigenous breeds, including Morada Nova, Santa Inês, and Pelibuey sheep, exhibit enhanced scrotal testicular thermoregulation and can maintain relatively stable semen quality under high ambient temperatures [4,18,19,20,21]. These adaptive advantages are associated with physiological traits such as increased sweat gland activity, efficient skin vasodilation, and effective respiratory heat dissipation, as well as underlying genetic differences related to stress response pathways. In this context, the differential cellular sensitivity observed between Hu and Wugu-Hu rams in the present study is consistent with previously reported breed-dependent variation in heat tolerance among sheep. Such variation likely arises from a combination of physiological adaptation and molecular regulation, including differences in immune modulation and stress-responsive gene expression, reinforcing the importance of breed background in determining testicular resilience to thermal stress.

### 4.2. Endocrine Stability Under Mild Heat Stress

The maintenance of relatively stable testicular concentrations of FSH and LH following 3 days of mild heat exposure suggests that gross disruption of hypothalamic–pituitary–gonadal (HPG) axis regulation was unlikely under the experimental conditions used. Both FSH and LH are regulated by the HPG axis. They are released from the pituitary gland and act on Sertoli cells and Leydig cells in the testes, respectively [5,22]. These results suggest that germ cell impairment may have resulted from direct local effects of heat rather than altered endocrine hormones mediated via hypothalamic control.

Testosterone, another key hormone in the testes, is secreted by Leydig cells and directly influences spermatogenesis by acting on germ cells and Sertoli cells [23]. In accordance with the unchanged testicular LH concentration, the stability of testosterone also implies that Leydig cell function was maintained, and that the observed loss of germ cells likely resulted from events within the seminiferous tubules rather than endocrine deficiency. This distinction is important because it indicates that the early testicular cell events triggered by mild heat stress are not driven by local testicular hormonal environment imbalance. However, tissue hormone concentrations alone cannot fully reflect systemic endocrine regulation. Future investigations combining testicular tissue measurements with serum concentrations of LH, FSH, and testosterone would allow a more comprehensive evaluation of endocrine responses to heat stress.

### 4.3. Cellular Alterations in the Seminiferous Epithelium

Although testicular hormone levels remained stable, notable structural damage was observed in the seminiferous tubules, particularly in Wugu-Hu rams. The more obvious effects in Wugu-Hu rams were indicated by breed-specific differences in cellular susceptibility to heat stress. The significant decrease in the total number of cells per seminiferous tubule in Wugu-Hu rams further supports the conclusion that the thermal threshold for structural integrity is lower in Wugu-Hu rams, which can lead to compromised microenvironments for germ cell development. This loss of cells is consistent with previous evidence indicating a significant increase in apoptotic cell numbers in Wugu-Hu sheep and other mammals [16,24,25].

The key function of Sertoli cells is to maintain the seminiferous structure and provide nutritional support to germ cells [26]. Quantitative immunofluorescence revealed that the number of SOX9-positive Sertoli cells remained stable after treatment, indicating that Sertoli cells were not preferentially lost under the applied heat stress conditions. This observation is consistent with previous in vitro studies, which report that Sertoli cells tolerate moderately high temperatures but exhibit functional changes rather than apoptosis [27]. In our study, scrotal temperature increased by about 3 °C, based on sustained measurements obtained using an established experimental model with continuous temperature monitoring as described previously, which may have been insufficient to reach the critical threshold for inducing Sertoli cell death. Therefore, the morphological disorganization observed in Wugu-Hu rams likely reflects secretory alterations in Sertoli cells rather than direct cell loss. This may be because the observed seminiferous tubule disorganization occurred in the absence of detectable Sertoli cell loss, and changes in Sertoli cells junctional protein expression or cytokine secretion are closely related to seminiferous tubule structure [28]. Further studies on ovine Sertoli cells secretory markers and oxidative stress parameters could clarify this hypothesis.

Spermatocytes represent another stage of spermatogenesis highly susceptible to heat stress, as they undergo active mitosis and meiosis [25,29]. Heat stress can compromise their survival by inducing DNA damage, chromosomal abnormalities, and cell cycle arrest [10,30]. However, it should be noted that most of the mechanistic evidence supporting these processes is derived from rodent models, and the extent to which these mechanisms are conserved in sheep remains to be fully established. In the present study, the number of spermatocytes was indirectly estimated due to the lack of well validated antibodies with sufficient specificity to reliably identify and quantify all spermatocyte stages in rams. Accordingly, these estimates are provided for descriptive purposes only and should be interpreted as reflecting relative trends rather than precise quantitative differences.

Spermatids are the next stage after meiosis. Both Hu and Wugu-Hu sheep showed a significant reduction in spermatid numbers following mild heat stress, with a larger reduction in Wugu-Hu rams. This reduction may reflect multiple, non-mutually exclusive processes. On the one hand, the decrease in spermatid cell number was caused by the enhanced mechanisms of apoptosis in spermatids [16]. There was also evidence that long-term exposure to scrotal hyperthermia in human ejaculated spermatozoa might have led to heat stress, which directly compromised spermatid viability by inducing mitochondrial dysfunction, ultimately impairing cellular function and survival [31]. However, because the present study examined short-term mild heat stress in ovine testicular tissue, the relevance of this comparison is supportive rather than direct. On the other hand, it is also possible that the spermatocytes were affected, so some spermatocytes could not successfully develop into spermatids, which ultimately led to the apoptosis of spermatocytes and a reduction in the number of spermatids. Based on the change in expression of the meiosis related genes of Wugu-Hu sheep reported in our previous study, we speculate that the spermatocytes underwent disrupted meiotic progression, increased apoptosis, or impaired differentiation and maturation processes [16,32,33], thereby contributing to the reduction in spermatid numbers. In contrast, spermatogonia showed breed-specific responses to mild heat stress. While no significant change was observed in Hu rams, a significant decrease was detected in Wugu-Hu rams, indicating that early germ cells in Wugu-Hu sheep may also be susceptible to heat stress. Nevertheless, even in the absence of marked numerical changes in Hu rams, functional impairment of spermatogonia cannot be excluded. Previous studies have demonstrated that mild heat stress can suppress spermatogonia self-renewal and proliferative capacity without inducing immediate apoptosis [29,34]. Thus, stable cell numbers may mask subtle functional deficits that could influence long-term recovery of spermatogenesis following thermal insult.

The persistence of spermatogonia, together with the selective loss of spermatocytes and spermatids, supports the idea of a cellular compensation mechanism in which early germ cells survive and later restore spermatogenic activity after temperature returns to normal. This selective removal of damaged cells may represent an adaptive strategy that preserves sperm quality after a mild thermal insult at the expense of a temporary reduction in sperm cell numbers, consistent with previous findings [16]. It should be noted that apoptosis was not directly assessed using TUNEL or other cell-type-specific assays in this study. Therefore, interpretations regarding apoptotic processes are based on indirect evidence derived from changes in germ cell numbers. Future studies incorporating such approaches will be necessary to directly validate these inferences.

### 4.4. Transcriptomic Insights into Breed-Specific Responses

In our previous study Chen et al., 2025 [16], transcriptomic analyses within each breed (treatment vs. control) revealed heat stress-induced alterations associated with apoptosis, meiotic disruption, and germ cell quality control mechanisms. In contrast, the present analysis focuses on differences between breeds under comparable conditions, which likely reflect inherent baseline variation rather than direct responses to heat stress alone.

The transcriptomic comparisons between the two sheep breeds identified 854 differentially expressed genes between Wugu-Hu and Hu rams, with functional enrichment suggesting differences in immune-related processes, including immune response, antigen presentation, and extracellular signalling. These differences are more likely to reflect breed-specific variation in testicular immune regulation rather than direct transcriptional responses to heat stress. Such variation may indicate differences in the immune microenvironment of the testes between breeds. As the testis is an immune-privileged organ, subtle changes in immune-related gene expression may influence the balance between immune tolerance and inflammatory responses under stress conditions [35]. Notably, genes associated with antigen processing and B-cell receptor signalling were more highly expressed in Wugu-Hu rams, which may suggest a relatively more active immune profile. This could potentially contribute to differences in cellular responses under heat stress; however, this interpretation remains speculative and requires further experimental validation [36,37].

The present study also included a KEGG pathway analysis; however, no pathways were significantly enriched. The absence of significantly enriched KEGG pathways may be attributed to the relatively small number of differentially expressed genes, the applied statistical thresholds, or the possibility that breed-specific differences are distributed across multiple biological processes rather than concentrated in specific signalling pathways.

### 4.5. Protein Interaction Network Analysis

The PPI network analysis between Wugu-Hu ram and Hu ram shows breed-specific molecular characteristics. The ribosomal proteins, including RPL6, RPL9, RPL36, RPL9-4, RPS3A, and MRPS12, emerged as core hub proteins, indicating the fundamental differences in translational capacity and protein synthesis between the two breeds. It is worth noting that the differences in the protein ISG15 exhibited the highest degree of connectivity within this network. ISG15 is a gene that encodes a small protein capable of forming covalent bonds with target proteins, a process known as ISGylation, which is involved in antiviral responses and cellular stress reactions in sheep testes [38]. Its high connectivity suggests substantial differences in immune function and stress response capacity between the two sheep breeds, which may underlie their distinct molecular responses to heat stress. However, it should be noted that the PPI network analysis performed in this study is based on computational predictions and does not provide direct evidence of functional interactions. Therefore, the identification of ribosomal proteins and ISG15 as highly connected nodes should be interpreted as indicative of potential regulatory relevance rather than definitive functional roles.

## 5. Conclusions

In conclusion, 3 days of mild scrotal insulation in Wugu-Hu and Hu rams did not disrupt endocrine stability but caused breed-dependent testicular responses. Wugu-Hu rams exhibited greater sensitivity to mild heat stress, particularly in Sertoli cells and late-stage germ cells. The germ cell loss may occur during the transition from spermatocytes to spermatids. Transcriptomic and PPI analyses revealed that the differences in immune regulation and translational activity may underlie different adaptive capacities of the two breeds. Together, these findings advance the understanding of cellular compensation and molecular regulation during testicular heat stress in different sheep breeds.

## Figures and Tables

**Table 1:** Descriptive estimation of spermatocyte numbers per seminiferous tubule in Hu and Wugu-Hu rams before and after scrotal insulation.

	Wugu-Hu Ram	Hu Ram
Pre-Insulation	81.79 ± 88.09	66.37 ± 79.62
Post-Insulation	81.3 ± 75.53	87.86 ± 68.29

Note: Data are presented as mean ± SD (*n* = 100). Spermatocyte values were indirectly derived from averaged counts of total cells, spermatogonia, spermatids, and Sertoli cells. These data are provided for descriptive purposes only, and no statistical comparison was applied.

## Data Availability

The datasets generated and analyzed during the current study are available from the corresponding author upon reasonable request.

## Abbreviations

The following abbreviations are used in this manuscript:

BSA	bovine serum albumin
CC	cellular component
DAPI	4′,6-Diamidino-2-phenylindole
DEG	differentially expressed gene
ELISA	enzyme-linked immunosorbent assay
FSH	follicle-stimulating hormone
GO	Gene Ontology
HPG axis	hypothalamic-pituitary-gonadal axis
IHC	immunohistochemistry
LH	luteinising hormone
MF	molecular function
PBS	phosphate-buffered saline
PNA	peanut agglutinin
PPI	protein–protein interaction
RNA-seq	RNA sequencing
